# Body composition as a predictor of oncological outcome in patients with non-muscle-invasive bladder cancer receiving intravesical instillation after transurethral resection of bladder tumor

**DOI:** 10.3389/fonc.2023.1180888

**Published:** 2023-08-10

**Authors:** Liang-Kang Huang, Yu-Ching Lin, Hai-Hua Chuang, Cheng-Keng Chuang, See-Tong Pang, Chun-Te Wu, Ying-Hsu Chang, Kai-Jie Yu, Po-Hung Lin, Hung-Cheng Kan, Yuan-Cheng Chu, Wei-Kang Hung, Ming-Li Hsieh, I-Hung Shao

**Affiliations:** ^1^ Division of Urology, Department of Surgery, Linkou Chang Gung Memorial Hospital, Taoyuan, Taiwan; ^2^ Department of Medical Imaging and Intervention, Chang Gung Memorial Hospital, Keelung and Chang Gung University, Taoyuan, Taiwan; ^3^ Department of Family Medicine, Linkou Chang Gung Memorial Hospital, Taoyuan, Taiwan; ^4^ Department of Urology, New Taipei Municipal TuCheng Hospital, Chang Gung Memorial Hospital and Chang Gung University, New Taipei, Taiwan; ^5^ College of Medicine, Chang Gung University, Taoyuan, Taiwan

**Keywords:** bladder cancer, non-muscle-invasive bladder cancer, sarcopenia, obesity, body composition, transurethral resection, intravesical instillation

## Abstract

**Introduction:**

Body status, categorized as sarcopenia or obesity and assessed using body mass index and body composition, affects the outcome of bladder cancer patients. However, studies comparing disease progression, recurrence, or overall survival in patients with non-muscle-invasive bladder cancer (NMIBC) with different body compositions are lacking. Therefore, we conducted a retrospective study to identify the impact of body composition, sarcopenia, and obesity on the oncological prognosis of patients with NMIBC who underwent transurethral resection of bladder tumor (TURBT) with Bacillus Calmette-Guerin (BCG) intravesical instillation (IVI).

**Methods:**

Patients with NMIBC who had undergone TURBT with adjuvant IVI with BCG from March 2005 to April 2021 were included. Body composition parameters were evaluated using computed tomography images of the third lumbar vertebrae and further categorized by sarcopenia and obesity. Oncological outcomes including recurrence-free survival (RFS), progression-free survival, and overall survival (OS) after treatment were analyzed.

**Results:**

A total of 269 patients were enrolled. Subcutaneous adipose tissue (SAT) density was a significant predictor of RFS, whereas psoas muscle density was a significant predictor of OS in the multivariate analysis. Patients with sarcopenia but without obesity tolerated significantly fewer BCG IVIs than patients without sarcopenia or obesity. Patients with sarcopenia had poorer RFS and OS than those without sarcopenia. In contrast, patients with obesity had better OS than those without obesity.

**Discussion:**

Body composition parameters, including SAT density and psoas muscle density, emerged as significant predictors of OS and RFS, respectively. Hence, our findings indicate that body composition is a helpful measurement to assess the oncological outcomes of patients with NMIBC.

## 1 Introduction

Bladder cancer (BC) is the 10^th^ most common cancer worldwide. BC is divided into two categories: non-muscle-invasive bladder cancer (NMIBC) and muscle-invasive bladder cancer (MIBC). The majority of the patients are initially diagnosed with NMIBC, and the 5-year survival rate of NMIBC is 90–98%, whereas only a 35–40% survival rate is reported for those with MIBC. Standard treatment of NMIBC includes transurethral resection of bladder tumor (TURBT) and intravesical instillation (IVI) with chemotherapy or immunotherapy (1). In contrast to MIBC, the most distinct treatment goal of NMIBC is to both spare the bladder and achieve oncological control. However, the strategy of sparing the bladder to maintain the quality of life could lead to a high risk of local recurrence. Thus, high 5-year recurrence (28.3–51.7%) and progression rates (4.6–19.8%) have been reported in the NMIBC patient population (2). Despite receiving IVI, a notable proportion of patients with NMIBC may still experience disease recurrence or progression to MIBC, which could lead to poor survival outcomes. Various studies have identified the risk factors for the recurrence of NMIBC after treatment, namely multifocal tumors larger than 3 cm, high tumor grade, or carcinoma *in situ* (3).

Among various risk factors, high body mass index (BMI) is also an independent risk factor for BC (4). BMI is simple, easy to measure, and is currently the most commonly used index to evaluate nutritional or metabolic status. It is simply expressed as the ratio of weight to height but does not distinguish between muscle and fat tissue (5). Meanwhile, body composition, which compensates for the limitations of BMI, is used to describe the percentage of fat, bone, and muscle in a human body.

Based on BMI and body composition, body status can be further categorized as sarcopenia and obesity, which has been reported to have a marked impact on the outcome of patients with BC. A meta-analysis conducted by Guo et al., which included patients who had undergone surgery for urological cancers, showed that sarcopenia had a worse impact on both overall and disease-specific survival (6). Furthermore, several authors have reported negative survival outcomes in patients with BC with sarcopenia after neoadjuvant chemotherapy and radical cystectomy (RC) (7, 8).

Obesity represents a type of body composition and may increase the risk of BC. Adiposity is connected with alterations in sex hormone metabolism, adipokine pathways, and inflammatory processes (9, 10). Although obesity is associated with increased incidence and poor prognosis in some cancers, including BC, a positive impact has been observed when patients with high BMI receive systemic immunotherapy with checkpoint inhibitors, resulting in improved outcomes (11). The phenomenon is sometimes known as the “obesity paradox” (12). There are conflicting opinions regarding the influence of obesity on BC prognosis.

Many studies have been conducted to determine the impact of sarcopenia or obesity on patients with MIBC. To the best of our knowledge, no study has compared disease progression, recurrence, or overall survival in patients with NMIBC with different body compositions. This study aimed to assess the impact of body composition, sarcopenia, and obesity on the oncological prognosis of patients with NMIBC.

## 2 Materials and methods

### 2.1 Patients

We retrospectively included patients with NMIBC who underwent TURBT with adjuvant Bacillus Calmette-Guerin (BCG) IVI from March 2005 to April 2021. All patients fulfilled the criteria of intermediate- or high-risk group classification, and all patients were newly diagnosed with NMIBC and were treatment naïve, with no recurrence. The medical charts and computed tomography (CT) images of all the patients were collected from a single tertiary medical center. Patients with incomplete data or images were excluded. This study was approved by the Institutional Review Board of the Chang Gung Medical Foundation (IRB no. 202100259B0) and followed the ethical principles outlined in the Declaration of Helsinki. The requirement for informed consent was waived owing to the retrospective study design.

### 2.2 Data collection

According to the American Joint Committee on Cancer, NMIBC is defined as carcinoma *in situ* (Tis), tumors confined to the mucosa (Ta), or tumors invading the lamina propria (T1). All preoperative patient information, including height, weight, underlying diseases, and Eastern Cooperative Oncology Group performance status, were recorded. Information on tumor-related parameters, such as tumor histologic type, primary tumor numbers, and whether the patient had any cancer other than BC or upper tract urothelial carcinoma, were also collected. Recurrence-free survival (RFS), progression-free survival (PFS), and overall survival (OS) were documented as endpoints. Survival status was documented according to the patient’s chart or condition at the last follow-up. RFS was measured from disease-free time to the time of recurrence in the bladder, detected using cystoscopy or follow-up imaging. Progression was defined as Tis, Ta, or T1 to a higher stage of T2 to T4, any N or any M. PFS was defined as disease-free time to the time of progression according to the imaging or pathology report during follow-up. OS was assessed from the first TURBT until death or the last follow-up.

### 2.3 Image analyses

For the assessment of body composition, we obtained CT images of patients before surgery as the benchmark and selected four images at the level of the third lumbar vertebra (L3) for subsequent analyses. We then assessed body composition based on CT images. CT images were analyzed, and five indices were derived from the image, including subcutaneous adipose tissue (SAT), visceral adipose tissue (VAT), all skeletal muscles (MUSCLE), paraspinal muscle (PARA), and psoas muscle (PSOAS). All indices were calculated with the area in square centimeters (cm^2^) and radiation attenuation in the Hounsfield unit (HU).

Body composition area is a measurement of the axial slice close to the inferior aspect of the vertebral body. The height adjustment area was computed as the body composition area divided by the square of height, which normalized the area of adipose tissue or muscle for height. Radiation attenuation is a measurement of the fat content of adipose tissue or muscle and is correlated with physical function. Radiation attenuation was calculated as the mean HU value of all pixels within the intended area of adipose tissue or muscle (13).

### 2.4 Sarcopenia and obesity

According to the European Working Group on Sarcopenia in Older Adults, the cutoff value of sarcopenia is set at two standard deviations below the mean of the healthy young adult population (14). According to Derstine et al., the cutoff values of skeletal muscle area and radiation attenuation at the L3 level of sarcopenia were 34.4 cm^2^/m^2^ and 34.3 HU, respectively, in women and 45.4 cm^2^/m^2^ and 38.5 HU, respectively, in men (13). In this study, we also considered adipose tissue and muscle, and patients with both indices below the cutoff values were included in the sarcopenia group.

BMI is a widely recognized surrogate marker for adiposity for patients of any age, calculated as weight divided by the square of height (kg/m^2^). Obesity is defined as BMI greater than 30 kg/m^2^. However, in Taiwan, the Ministry of Health and Welfare defines obesity as a BMI greater than 27 kg/m^2^. Therefore, in this study, patients with a BMI greater than 27 kg/m^2^ were included in the obese group (15).

### 2.5 Statistical analyses

Pearson’s correlation test was used to analyze the association between sarcopenia and obesity in patients with NMIBC according to age at diagnosis, number of BCG IVIs, and cancer stage. The correlation between the prognostic factors and endpoints was assessed using Cox proportional hazards models and logistic regression test. Finally, Kaplan–Meier survival test was used to determine the correlation between sarcopenia/obesity and survival benefits. All tests were two-sided, and p-values <0.05 were statistically significant. All statistical analyses were performed using IBM SPSS Statistics for Mac, version 25 (IBM Corp, Armonk, NY, USA).

## 3 Results

**Figure 1:**
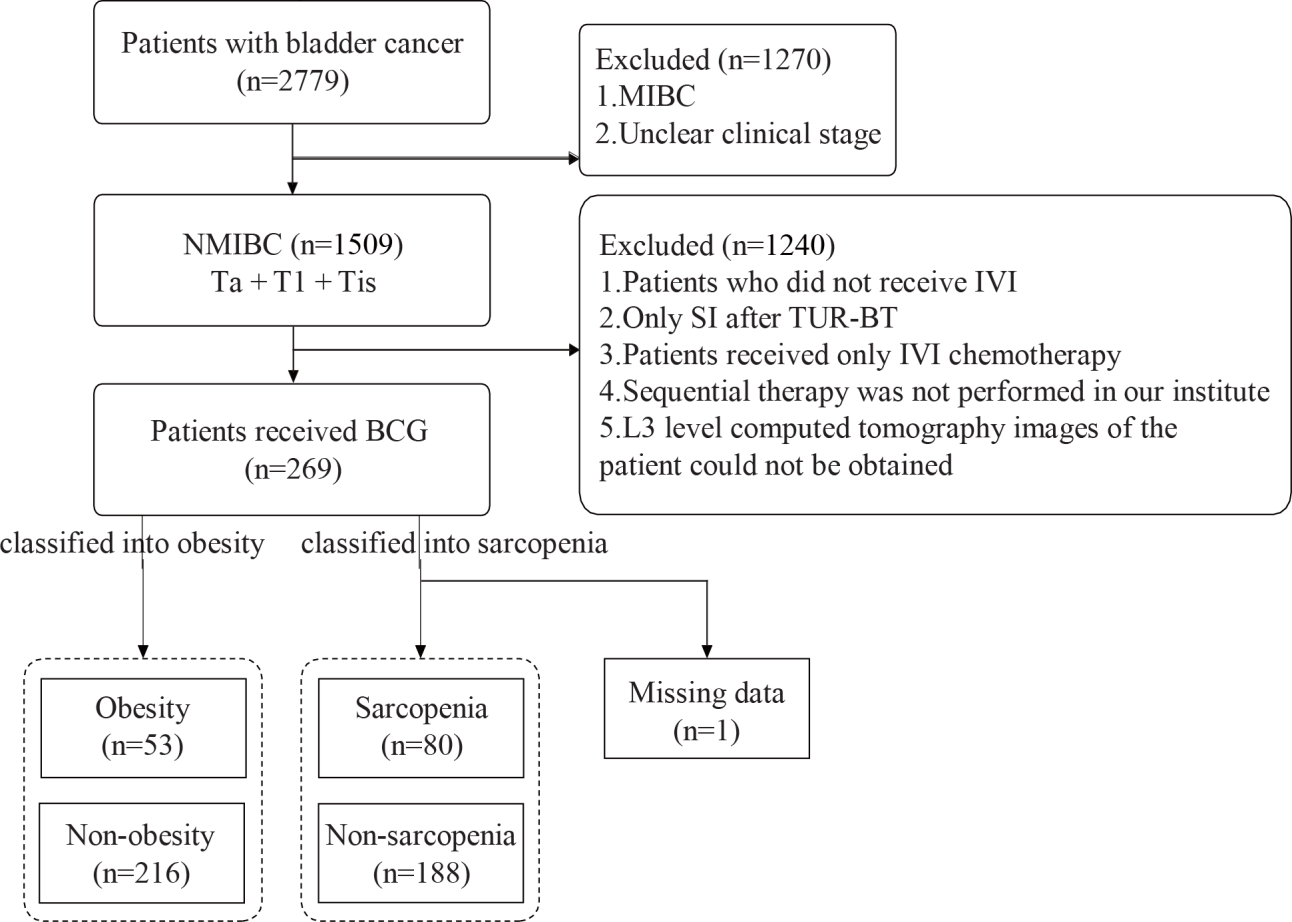
Flowchart of patient selection. MIBC, muscle-invasive bladder cancer; NMIBC, non-muscle-invasive bladder cancer; Tis, carcinoma in situ; Ta, tumors confined to the mucosa; Tl, tumors invading the lamina propria; IVI, intravesical instillation; TURBT, transurethral resection of bladder tumor; CT, computed tomography.

We initially restrospectively collected 2779 bladder cancer patients. In total, 269 patients were enrolled in this study. The flowchart was showed in **Figure 1**. Among them, 208 (77.3%) were men and 61 (22.7%) were women. The mean age of the patients at diagnosis was 66.41 years. All patients were diagnosed with NMIBC, and most had Ta or T1 disease, accounting for 44.6% and 50.9%, respectively. According to the CT images and BMI data obtained at the time of the initial diagnosis of NMIBC, 80 patients had sarcopenia, whereas 53 had obesity, accounting for 29.7% and 19.7%, respectively. A total of 111 patients (41.3%) experienced recurrence, whereas 68 (25.3%) experienced progression. The other general characteristics and tumor-related parameters are presented in **Table 1**.

**Table 1:** Patients’ general characteristics and body composition indices (n = 269).

Patients’ general characteristics	
Sex	
Male	208 (77.30)
Female	61 (22.70)
Age at diagnosis (years)	66.41±11.46
Height (cm)	161.63±8.23
Weight (kg)	64.23±11.66
BMI (kg/m^2^)	24.49±3.52
Smoking	
Yes	100 (37.2)
No	169 (62.8)
Hypertension	
Yes	147 (54.6)
No	122 (45.4)
Diabetes mellitus	
Yes	58 (21.6)
No	211 (78.4)
ECOG	
0	191 (71.0)
1	69 (25.7)
2	9 (3.3)
Sarcopenia	
Yes	80 (29.70)
No	188 (69.90)
Missing	1 (0.4)
Obesity	
Yes	53 (19.7)
No	216 (80.3)
Tumor-related parameters	
Cancer stage	
Tis	12 (4.5)
Ta	120 (44.6)
T1	137 (50.9)
Type of histology	
Low grade	68 (25.3)
High grade	190 (70.6)
Unknown	11 (4.1)
Tumor number	
Single	118 (43.9)
Multiple	151 (56.1)
Cancer other than BC and UTUC	
Yes, before BC	13 (4.8)
Yes, after BC	38 (14.1)
No	213 (79.2)
Unknown	5 (1.9)
Recurrence	
Yes	111 (41.3)
No	158 (58.7)
Progression	
Yes	68 (25.3)
No	201 (74.7)
Survival status	
Alive	170 (63.2)
Dead	99 (36.8)
BCG IVI (times)	10.7±5.1
Area	
SAT (cm^2^)	115.9±49.7 [14.7 to 327.3]
VAT (cm^2^)	139.8±70.4 [4.3 to 415.2]
MUSCLE (cm^2^)	120.4±27.2 [64.1 to 200.3]
PARA (cm^2^)	48.5±11.6 [15.3 to 80.0]
PSOAS (cm^2^)	14.5±4.9 [5.6 to 28.9]
Height adjustment index	
SAT (HU)	44.7±19.6 [6.5 to 131.1]
VAT (HU)	53.4±26.4 [1.7 to 152.5]
MUSCLE (HU)	45.8±8.6 [26.5 to 75.4]
PARA (HU)	18.5±3.8 [6.8 to 29.3]
PSOAS (HU)	5.5±1.7 [2.1 to 12.1]
Radiation attenuation	
SAT	−113.7±106.2 [−1425.7 to −31.0]
VAT	−95.2±11.0 [−163 to [−52]
MUSCLE	31.5±9.0 [−8.05 to 48.2]
PARA	34.5±10.3 [−15 to 51.7]
PSOAS	39.6±7.2 [10.3 to 56.1]

Values are presented as n (%), mean±standard deviation, or mean±standard deviation [range].

ECOG, Eastern Cooperative Oncology Group performance status scale; BC, bladder cancer; UTUC, upper urinary tract cancer; BCG IVI, Bacillus Calmette-Guerin intravesical instillation; SAT, subcutaneous adipose tissue; HU, Hounsfield unit; VAT, visceral adipose tissue; MUSCLE, all skeletal muscles; PARA, paraspinal muscles; PSOAS, psoas muscle.

Based on the CT images for staging, the body composition areas of the five parameters of the SAT, VAT, MUSCLE, PARA, and PSOAS were 115.9, 139.8, 120.4, 48.9, and 14.5 cm^2^, respectively. The height adjustment areas for the five parameters were 44.7, 53.4, 45.8, 18.5, and 5.5 cm^2^/m^2^. The radiation attenuation values of each part were −113.7, −95.2, 31.5, 34.5, and 39.6 HU, respectively. Details of the remaining body composition analyses are presented in **Table 1**.

After analyzing univariate and multivariate Cox proportional hazards models, we found that age at diagnosis and BMI were correlated with OS (p < 0.001 and p = 0.018, respectively). The RFS and PFS differed significantly according to cancer stage (p < 0.001 and p = 0.001, respectively). In addition, we demonstrated a significant effect of the number of times patients received BCG IVI on the RFS, PFS, and OS of patients (p < 0.001, p < 0.001, and p = 0.025, respectively).

As for the impact of body composition parameters on the RFS, PFS, and OS, we found that radiation attenuation by SAT had a statistically significant impact on the RFS (hazards ratio [HR] = 0.998, 95% confidence interval [CI] = 0.99–1.00, p = 0.011), whereas radiation attenuation by PSOAS had no effect on the RFS (HR = 0.978, 95% CI = 0.96–1.00, p = 0.053) and a significant impact on the OS (HR = 0.948, 95% CI = 0.90–1.00, p = 0.044). Radiation attenuation by MUSCLE and PARA also had notable effects on the OS. In contrast, the height adjustment areas of SAT, MUSCLE, and PSOAS had a significant impact on the OS (p = 0.03, p = 0.03, and p = 0.04, respectively). However, we were unable to identify a single parameter as a prognostic factor for patients with NMIBC in this study. Age, cancer stage, the number of times BCG IVI was received, radiation attenuation, and height adjustment area had an effect on the RFS, PFS, and OS. The rest of the Cox proportional hazards model results are presented in **Table 2**.

**Table 2:** Summary of the univariate and multivariate Cox regression analyses.

Recurrence-free survival									
	Unit	Mean	SD	Univariate analysis			Multivariate analysis		
				HR	95% CI	p-Value	HR	95% CI	p-Value
Age at diagnosis	years	66.4	11.46	1.016	0.99–1.03	0.073			
BMI	kg/m^2^	24.5	3.52	0.966	0.92–1.02	0.193			
Cancer stage				1.646	1.23–2.21	0.001*	1.861	1.38–2.52	<0.001**
BCG IVI	times	10.7	5.1	0.902	0.88–0.94	<0.001**	0.892	0.86–0.93	<0.001**
Radiation attenuation									
SAT	HU	−114	106.2	0.998	0.99–0.99	0.001*	0.998	0.99–0.99	0.011*
VAT	HU	−95	11.0	0.998	0.98–1.02	0.827			
MUSCLE	HU	31.5	9.0	0.99	0.97–1.01	0.329			
PARA	HU	34.5	10.3	0.998	0.98–1.02	0.803			
PSOAS	HU	39.6	7.2	0.97	0.95–0.99	0.012*	0.978	0.96–1.00	0.053
Height adjustment index									
SAT	cm^2^/m^2^	44.7	19.6	0.997	0.99–1.01	0.482			
VAT	cm^2^/m^2^	53.4	26.4	1.001	0.99–1.01	0.742			
MUSCLE	cm^2^/m^2^	45.8	8.6	0.979	0.96–1.00	0.055			
PARA	cm^2^/m^2^	18.5	3.8	0.972	0.93–1.02	0.234			
PSOAS	cm^2^/m^2^	5.5	1.7	0.935	0.84–1.04	0.230			

Progression-free survival									
	Unit	Mean	SD	Univariate analysis			Multivariate analysis		
				HR	95% CI	p-Value	Exp(B)	95% CI	p-Value
Age at diagnosis	years	66.4	11.46	1.012	0.99–1.03	0.306			
BMI	kg/m^2^	24.5	3.52	0.956	0.90–1.02	0.183			
Cancer stage				1.961	1.36–2.85	<0.001**	2.269	1.55–3.33	0.001*
BCG IVI	times	10.7	5.1	0.936	0.89–0.98	0.009*	0.918	0.87–0.97	<0.001**
Radiation attenuation									
SAT	HU	−114	106.2	1	0.99–1.00	0.967			
VAT	HU	−95	11.0	1.013	0.99–1.00	0.188			
MUSCLE	HU	31.5	9.0	0.995	0.97–1.02	0.701			
PARA	HU	34.5	10.3	1.001	0.98–1.03	0.904			
PSOAS	HU	39.6	7.2	0.976	0.95–1.01	0.129			
Height adjustment index									
SAT	cm^2^/m^2^	44.7	19.6	0.995	0.98–1.01	0.427			
VAT	cm^2^/m^2^	53.4	26.4	0.994	0.99–1.00	0.228			
MUSCLE	cm^2^/m^2^	45.8	8.6	0.983	0.96–1.01	0.238			
PARA	cm^2^/m^2^	18.5	3.8	1.002	0.94–1.06	0.96			
PSOAS	cm^2^/m^2^	5.5	1.7	0.957	0.83–1.10	0.539			

Overall survival									
	Unit	Mean	SD	Univariate analysis			Multivariate analysis		
				Exp(B)	95% CI	p-Value	Exp(B)	95% CI	p-Value
Age at diagnosis	years	66.4	11.46	1.056	1.04–1.08	<0.001**	1.044	1.02–1.07	<0.001**
BMI	kg/m^2^	24.5	3.52	0.9	0.85–0.95	<0.001**	0.91	0.84–0.98	0.018*
Cancer stage				1.274	0.92–1.76	0.14			
BCG IVI	times	10.7	5.1	0.935	0.90–0.97	0.001*	0.949	0.91–0.99	0.025*
Radiation attenuation									
SAT	HU	-114	106.2	1	0.99–1.00	0.968			
VAT	HU	-95	11.0	1.016	0.99–1.03	0.062			
MUSCLE	HU	31.5	9.0	0.964	0.95–0.98	0.001*	1.001	0.92–1.09	0.988
PARA	HU	34.5	10.3	0.971	0.96–0.99	<0.001**	1.016	0.96–1.07	0.582
PSOAS	HU	39.6	7.2	0.952	0.93–0.98	<0.001**	0.948	0.90–0.99	0.044*
Height adjustment index									
SAT	cm^2^/m^2^	44.7	19.6	0.988	0.98–0.99	0.03*	1	0.99–1.02	0.981
VAT	cm^2^/m^2^	53.4	26.4	0.998	0.99–1.01	0.632			
MUSCLE	cm^2^/m^2^	45.8	8.6	0.975	0.95–0.99	0.031*	1.016	0.98–1.06	0.434
PARA	cm^2^/m^2^	18.5	3.8	0.958	0.91–1.01	0.09			
PSOAS	cm^2^/m^2^	5.5	1.7	0.883	0.78–0.99	0.04*	1.062	0.88–1.28	0.523

SD, standard deviation; HR, hazards ratio; CI, confidence interval; BMI, body mass index; BCG IVI, Bacillus Calmette-Guerin intravesical instillation; SAT, subcutaneous adipose tissue; HU, Hounsfield unit; VAT, visceral adipose tissue; MUSCLE, all skeletal muscles; PARA, paraspinal muscle; PSOAS, psoas muscle; height-adjusted area, body composition area at the third lumbar (L3) vertebral level/height^2^

*p < 0.05, **p < 0.001. The significant values were showed as bold with * or bold with **.

In Pearson’s correlation analysis test, we found statistically significant correlations among sarcopenia or obesity, age at diagnosis, and the number of BCG IVIs. Patients with sarcopenia were diagnosed at an older age (r = 0.263, p < 0.001), whereas patients with obesity were diagnosed at a younger age (r = −0.155, p = 0.011; **Table 3**). In addition, patients with sarcopenia were found to receive fewer BCG IVIs (r = −0.178, p = 0.003) than patients without sarcopenia, whereas patients with obesity received more BCG IVIs than those without obesity (r = 0.126, p = 0.039; **Table 3**).

**Table 3:** Correlation matrix between sarcopenia/obesity and significant factors.

Parameters	Age at diagnosis	BMI	Number of times of BCG IVI	Cancer stage	Tumor grading	Obesity	Sarcopenia
Sarcopenia	0.263**	−0.227*	−0.178**	0.001	**−**0.40	−0.222**	1
	***0.000***	***0.000***	***0.003***	*0.992*	*0.516*	*0.000*	−
Obesity	−0.155*	0.723**	0.126*	0.090	0.073	1	−0.222**
	***0.011***	***0.000***	***0.039***	*0.141*	*0.230*	−	*0.000*

The numbers are the correlation coefficients, and the p-values are presented in italics. Statistically significant values are shown in bold type. BMI, body mass index; BCG IVI, Bacillus Calmette-Guerin intravesical instillation. *Correlation is significant at the 0.05 level. **Correlation is significant at the 0.01 level.

In all, 147 patients discontinued BCG IVIs during treatment: 52 patients discontinued owing to adverse events, three discontinued owing to BCG shortage, and the status was not documented in 92 patients. As per chi-square analysis, patients with sarcopenia had a higher risk of discontinuation of BCG IVIs owing to adverse events (p = 0.012).

**Figure 2:**
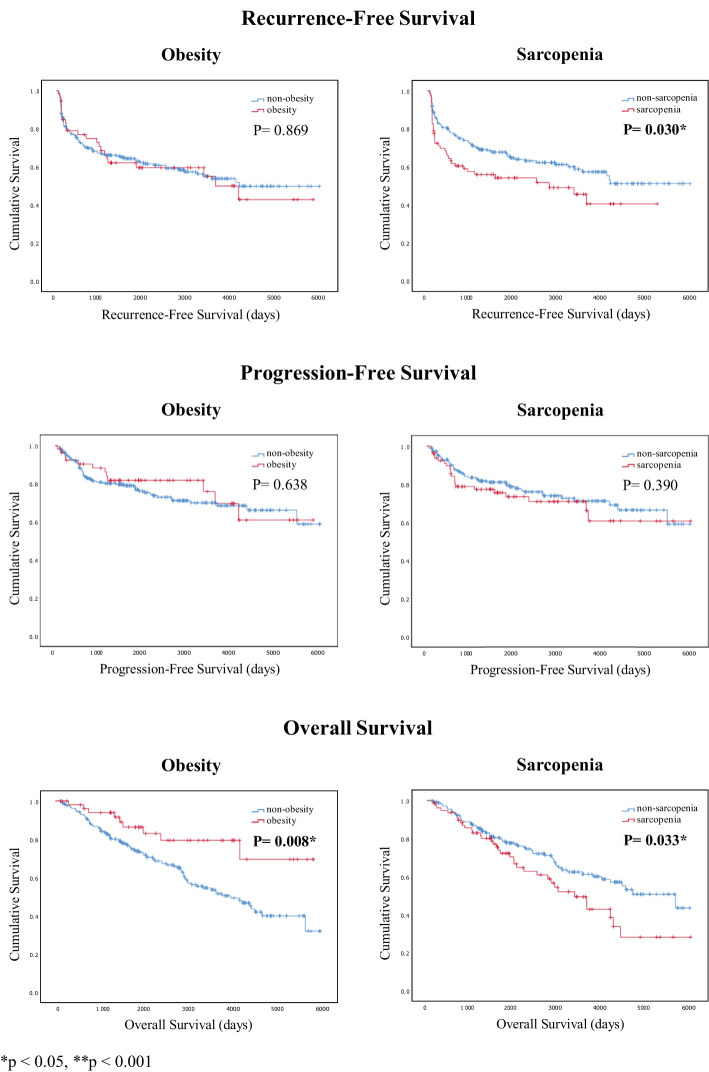
Kaplan–Meier survival curves of recurrence-free, progression-free, and overall survival analyses for patients with obesity and sarcopenia.

Finally, using the Kaplan–Meier survival test, we found that patients with sarcopenia had poorer RFS and OS (p = 0.030 and p = 0.033, respectively). In contrast, patients with obesity had better OS (p = 0.008). However, with regards to PFS, there was no significant difference between patients with sarcopenia and obesity (p = 0.390 and p = 0.638, respectively; **Figure 2**).

## 4 Discussion

This is the first study to demonstrate the impact of body composition on the oncological prognosis of patients with NMIBC treated with TURBT and BCG instillation. Body composition parameters, such as SAT density and psoas muscle density, were significant predictors of RFS and OS, respectively. Sarcopenia and obesity are simple indicators of lean and adipose tissue mass. Therefore, we classified sarcopenia according to the skeletal muscle index, using CT measurements (16) and also considered the results of Derstine et al. (13) and adipose tissue and muscle into account; patients with both indices below the cutoff values were included in the sarcopenia group. Approximately 30% of the patients were diagnosed with sarcopenia. The incidence was similar to that previously reported in patients with advanced BC (17).

Psutka et al. were the first to report that patients with sarcopenia and BC undergoing RC had poorer cancer-specific survival and OS than those without sarcopenia (18). In addition, Ornaghi et al. showed that patients with sarcopenia and BC had worse 5-year cancer-specific survival and OS than those without sarcopenia (19). Moreover, Mayr et al. showed an independent correlation between sarcopenia and increasing cancer-specific mortality (CSM) and all-cause mortality among patients receiving RC (20). Meanwhile, Hu et al. showed sarcopenia to be correlated with diminished cancer-specific survival and OS in patients with urothelial cancer (21). Lyon et al. also found that lower skeletal muscle mass after treatment with neoadjuvant chemotherapy, but before RC, had a markedly negative impact on CSM after surgery (22).

The exact underlying cause for fewer BCG IVIs received by patients with sarcopenia remains unknown. Loosen et al. demonstrated that sarcopenia negatively influences immune checkpoint therapy for cancer treatment. They linked sarcopenia to the activation of the immune system and systemic inflammation. This association resulted in impaired anti-cancer effects of immunotherapy (23).

However, as mentioned previously, BCG IVI is the recommended standard treatment for the prevention of recurrence and progression after TURBT in high-risk patients with NMIBC (1). Nonetheless, most patients with NMIBC treated with BCG IVI experience BCG toxicity, such as flu-like symptoms and cystitis, and some patients develop systemic tuberculosis infections (24, 25). In a previous study, nearly 2.9% of the patients with NMIBC discontinued immunotherapy owing to systemic or urinary BCG-related infection or intolerability (26). Meanwhile, different BCG strains such as TICE and RIVM may also impact the oncological outcomes of patients with BC (27). However, whether a different strain would result in a different type of toxicity remains unknown.

In our study, we noted that patients with sarcopenia were older and required fewer BCG IVIs than those who were not. Older patients usually have sarcopenia; moreover, their muscle mass decreases owing to poor nutrition, hormonal status, and lack of sports activities. Patients with sarcopenia usually have poor general health, and skeletal muscle deficiency may be correlated with the activation of the immune response (28). Thus, we hypothesized that patients with sarcopenia might have poorer tolerance to BCG IVI. As a result, treatment with few BCG IVIs in patients with sarcopenia will be less beneficial to them, further negatively influencing the OS and RFS.

Nevertheless, correlations between BMI and the risk of developing NMIBC or MIBC remain controversial. A 10% increase in the risk of BC development in patients with a high BMI was reported in three meta-analyses (9, 29, 30). In a discussion of prognosis among patients with NMIBC, recent meta-analyses showed that compared with patients who were not overweight or obese, patients who were overweight or obese had a markedly increased risk of cancer recurrence (31, 32). In another study, Kluth et al. found a negative correlation between recurrence and progression while comparing patients with and without obesity with high-grade T1 BC (33). High BMI was associated with a high risk of BC recurrence in a previous article (34). In addition to BMI and obesity, diet factors were also possibly related to the occurrence of BC. Multiple studies have illustrated the relationship between diet and risk of BC, including the cooking method, amount of meat intake, type of meat consumed, and temperature applied (35). Although many studies have mentioned that obesity is a negative risk factor for BC, these conclusions remain controversial.

Our study showed contradictory results to the studies above. Patients with obesity had better OS outcomes than those without obesity. This was echoed in the research conducted by Brooks et al. (36). They showed that elevated BMI was correlated with superior outcomes in patients with NMIBC who were treated with intravesical BCG immunotherapy. Arthuso et al. found that patients with advanced BC who were overweight or obese had a better prognosis within the first 5 years after RC (37). Obesity may be correlated with poorer outcomes in patients with NMIBC treated without BCG but is related to improved prognosis for those treated with intravesical immunotherapy (38, 39).

This can be explained by a few well-known mechanisms, namely increased activation of mitogenic pathways and increased levels of circulating inflammatory cytokines related to obesity (40). These factors may change the tumor microenvironment or systemic priming of BC, although BCG acts locally. This, in turn, may improve the oncological outcomes of BC (36). However, BCG IVI remains the gold standard treatment in terms of prevention of recurrence and progression in patients with NMIBC (1). The fact that patients with obesity received more BCG IVIs than those without obesity may have improved the OS of the patients with BC.

To our knowledge, previous studies aimed to determine the correlations among sarcopenia, cachexia, and nutritional status in patients with MIBC, regardless of whether the patients were receiving RC, neoadjuvant chemotherapy, or adjuvant chemotherapy. However, in our study, we aimed to determine the differences in the effect of sarcopenia and obesity on patients with BC. As shown in our results, patients with obesity had better OS than those without obesity, and patients with sarcopenia had worse OS and RFS than those without sarcopenia.

Our study had some limitations. First, we retrospectively collected data from approximately 300 patients. Second, the side effects and toxicity of BCG IVI were not analyzed in our study. More comprehensive data might help clarify the definite reasons for the discontinuation of BCG IVI. Third, we only calculated the results from some of the prognostic factors, such as the number of BCG IVIs, radiation attenuation, and height adjustment index. Nutritional and immune statuses, such as albumin level or neutrophil-to-lymphocyte ratio, were not assessed. Fourth, we showed significant differences in OS and RFS of patients with sarcopenia and OS of patients with obesity. We anticipate expanding our case numbers to determine if there is a correlation between body composition and PFS, which may better represent the independent role of sarcopenia in BC treatment. Fifth, all patients were recruited from a single center. Multicenter data collection is required for a thorough statistical analysis, and further prospective and larger series are needed to confirm our current findings. Sixth, OS was not an optimal oncological outcome for patients with NMIBC because NMIBC would progress to disseminated disease, thus impacting OS. However, OS was influenced by patient’s general condition, which may be correlated with sarcopenia or obesity. Thus, OS was still assessed as an endpoint in this study. Finally, there is currently no consensus regarding the definition of sarcopenia. Therefore, definitions differed across studies, and this may have led to a difference in the results between papers.

To our knowledge, this study is the first to demonstrate the impact of body composition on the oncological prognosis of NMIBC treated with TURBT and BCG instillation. Certain body composition parameters were found to be significant predictors of RFS and OS. Patients without sarcopenia and obesity can significantly tolerate more BCG IVIs, and this may result in favorable oncological outcomes. Among patients with NMIBC, the obese group had better OS than the non-obese group. Patients with sarcopenia had worse OS and RFS than those without sarcopenia. Further prospective research is required to validate the correlation between body composition and oncological outcomes in patients with NMIBC.

## Data availability statement

The original contributions presented in the study are included in the article/supplementary material. Further inquiries can be directed to the corresponding author.

## Ethics statement

All medical charts and computed tomography (CT) images of patients were reviewed and collected from a single tertiary medical center. Patients with incomplete data or images were excluded. This study was approved by the Institutional Review Board of the Chang Gung Medical Foundation. (IRB Number: 202100259B0), and followed the ethical principles outlined in the Declaration of Helsinki. The requirement for informed consent was waived owing to the retrospective study design.

## Author contributions

Conceptualization: L-KH and I-HS. Methodology: Y-CL. Software: W-KH. Validation: L-KH. Formal analysis: L-KH and I-HS. Investigation: L-KH. Resources: C-KC, S-TP, C-TW, Y-HC, K-JY, P-HL, I-HS, H-CK, Y-CC, L-KH and M-LH. Data curation: L-KH and I-HS: writing—original draft preparation: L-KH and W-KH. Writing—review and editing: H-HC and I-HS. Visualization: Y-CL. Supervision: C-TW and I-HS. Project administration: L-KH. All authors have read and agreed to the published version of the manuscript.
